# *Elizabethkingia* Intra-Abdominal Infection and Related Trimethoprim-Sulfamethoxazole Resistance: A Clinical-Genomic Study

**DOI:** 10.3390/antibiotics10020173

**Published:** 2021-02-09

**Authors:** Ling-Chiao Teng, Jiunn-Min Wang, Hsueh-Yin Lu, Yan-Chiao Mao, Kuo-Lung Lai, Chien-Hao Tseng, Yao-Ting Huang, Po-Yu Liu

**Affiliations:** 1 Section of Infectious Disease, Taichung Veterans General Hospital, Taichung 40705, Taiwan; ricecake1771@gmail.com (L.-C.T.); tedi3tedi3@hotmail.com (C.-H.T.); 2 Routine Laboratory, Taichung Veterans General Hospital, Taichung 40705, Taiwan; jmwang@vghtc.gov.tw; 3 Department of Computer Science and Information Engineering, National Chung Cheng University, Taichung 62102, Taiwan; mrfish2468@gmail.com; 4 Department of Emergency Medicine, Division of Clinical Toxicology, Taichung Veterans General Hospital, Taichung 40705, Taiwan; doc1385e@gmail.com; 5 National Defense Medical Center, School of Medicine, Taipei 11490, Taiwan; 6 Division of Allergy, Immunology and Rheumatology, Department of Internal Medicine, Taichung Veterans General Hospital, Taichung 40705, Taiwan; kllaichiayi@yahoo.com.tw; 7 Rong Hsing Research Center for Translational Medicine, National Chung Hsing University, Taichung 40227, Taiwan; 8 Ph.D. Program in Translational Medicine, National Chung Hsing University, Taichung 40227, Taiwan

**Keywords:** *Elizabethkingia anopheles*, trimethoprim-sulfamethoxazole, sequence alignment, whole genome sequencing

## Abstract

(1) *Background*: *Elizabethkingia* spp. is an emerging nosocomial pathogen which causes mostly blood stream infection and nosocomial pneumonia. Among *Elizabethkingia* species, *Elizabethkingia anophelis* is the major pathogen, but misidentification as *Elizabethkingia meningoseptica* is a common problem. *Elizabethkingia* also possesses broad antibiotic resistance, resulting in high morbidity and mortality of the infection. The aim of our study was to review *Elizabethkingia* intra-abdominal infections and investigate resistance mechanisms against TMP/SMX in *Elizabethkingia anophelis* by whole genome sequencing. (2) *Methods*: We retrospectively searched records of patients with *Elizabethkingia* intra-abdominal infection between 1990 and 2019. We also conducted whole genome sequencing for a TMP/SMX-resistant *Elizabethkingia anophelis* to identify possible mechanisms of resistance. (3) *Results*: We identified a total of nine cases of *Elizabethkingia* intra-abdominal infection in a review of the literature, including our own case. The cases included three biliary tract infections, three CAPD-related infection, two with infected ascites, and two postoperation infections. Host factor, indwelling-catheter, and previous invasive procedure, including surgery, play important roles in *Elizabethkingia* infection. Removal of the catheter is crucial for successful treatment. Genomic analysis revealed accumulated mutations leading to TMP/SMX-resistance in *folP*. (4) *Conclusions*: Patients with underlying disease and indwelling catheter are more susceptible to *Elizabethkingia* intra-abdominal infection, and successful treatment requires removal of the catheter. The emerging resistance to TMP/SMX may be related to accumulated mutations in *folP*.

## 1. Introduction

The genus *Elizabethkingia* was proposed by Kim in 2005 [1], and soon attracted attention due to nosocomial infection and broad antibiotic resistance. In the genus, *Elizabethkingia anophelis* was first discovered in 2011 from the midgut of mosquitoes in Africa [2]. With advances in identification, including 16s RNA sequencing and the availability of the MALDI-ToF system with new databases, *Elizabethkingia anophelis* was recognized as the dominant pathogen in *Elizabethkingia spp.*, rather than *Elizabethkingia meningoseptica* [3,4].

The first report of *Elizabethkingia anophelis* nosocomial outbreak was in a Singapore intensive care unit in 2012. Similar outbreaks were subsequently reported in hospitals in Wisconsin, USA, during 2015–2016 [5,6]. Most infections caused by *Elizabethkingia* species were blood stream infections, but pneumonia, septic arthritis, infected ascites, meningitis, and eye infection were also reported [6,7,8]. Recently, reports have emerged of intra-abdominal infections, such as postoperation infection, biliary tract infection, ascites infection, and CAPD infection. *Elizabethkingia anophelis* exhibit broad antibiotic resistance, including to most types of penicillin, cephazolin, carbapenem, aminoglycoside, and macrolide [9,10]. Fluoroquinolones, trimethoprim-sulfamethoxazole (TMP/SMX), and piperacillin/tazobactam were given as first-line therapy for *Elizabethkingia* infection, but emerging resistance has made treatment more challenging recently [9,10,11]. The development of sequencing technologies and genomic analyses are improving our understanding of the genetic background of resistance to antibiotics [12,13].

In this study, we present a case of *Elizabethkingia anophelis* infection with bacteremia and infected ascites and review the literature on *Elizabethkingia* infection with intra-abdominal infection. Whole genome sequencing and genome comparison were conducted to determine the genetic factors leading to resistance against TMP/SMX in *Elizabethkingia anophelis.*

## 2. Results

### 2.1. Case Report

A 69-year-old male who visited our hospital for severe pitting edema and dyspnea was admitted for autoimmune disease-related protein-losing enteropathy. A high-dose steroid was administered. Recurrent infection developed during the hospital course, including pneumonia, empyema, and several episodes of bacteremia.

*Elizabethkingia meningoseptica* bacteremia developed with fever, chills, dyspnea, and septic shock. Blood culture yielded two sets of *Elizabethkingia meningoseptica*, but re-identification by whole genome sequencing detected *Elizabethkingia anophelis.* Levofloxacin 750mg QD with TMP/SMX was administered. A survey for fever focus found ascite infection, favoring spontaneous bacterial peritonitis, and culture from ascites also yielded *Elizabethkingia anophelis.*

Follow-up blood culture after three days of antibiotic treatment still found a positive result. Repeat culture found a change in susceptibility. A new culture report showed *E. anophelis*, which was resistant to trimethoprim/sulfamethoxazole (TMP/SMX) but sensitive to piperacillin/tazobactam. Piperacillin/tazobactam was administered and TMP/SMX with levofloxacin was discontinued. There was no improvement in fever and progression of sepsis, and the patient expired about 10 days after piperacillin/tazobactam use, due to sepsis-related profound DIC and massive GI bleeding.

### 2.2. Reported Elizabethkingia Intra-Abdominal Infection in the Literature

A total of eight cases of *E. anophelis*- or *E. meningoseptica*-associated intra-abdominal infection were identified with detailed information. With the addition of our case, a total of nine cases are presented in this study [6,14,15,16,17,18] (Table 1 and Table 2).

Among the nine cases, there were two infected ascites, three CAPD peritonitis, three hepato-biliary infections, and one peritonitis after gynecologic procedure. Patients ages ranged from 8 to 89 years, and the female to male was 6:3. All patients had previous underlying disease, except for one with peritonitis after gynecologic procedure. The underlying diseases included type II diabetes mellitus, liver cirrhosis, end-stage renal disease, hypertension, and HCV infection. In two of nine (22%) cases of *Elizabethkingia*-related abdominal infection, they had previous procedure: liver transplantation and medical termination of pregnancy. Six of nine cases (66.7%) had indwelling catheter or stent inserted before the infection, with one PTBD, two biliary stent, and three CAPD tube. Most patients presented with fever and abdominal pain, but fever may be absent under immunosuppressant use.

Among the nine cases, three had *Elizabethkingia anophelis* infection and six had *Elizabethkingia meningoseptica* infection. Five positive cultures were yielded from blood culture, and bacteria growth from PTBD drainage in one case and from ascites or CAPD fluid in five cases was also identified. The pathogen was identified by Vitek 2 system in four cases, two by 16s rRNA sequencing, and one by MOLDI-ToF. Our case was re-identified by whole genome sequencing. The identification method was unavailable in the case with postliver transplantation infection from India.

Most cases with *Elizabethkingia* infection had broad antibiotic resistance but were susceptible to ciprofloxacin, levofloxacin, Cefoperazone/sulbactam, minocycline, TMP/SMX, piperacillin, piperacillin/tazobactam, and tigecycline. Most cases survived after antibiotic treatment, but for those with catheter—especially CAPD tube—infection were controlled only after removal of the CAPD tube.

#### Genomics Revealed Key Mutations Leading to TMP/SMX and Quinolone Resistance

The genome of *Elizabethkingia anophelis* SUE was sequenced by Nanopore and Illumina sequencing plateforms (Supplementary Table S1). The sequencing reads were assembled into a circular genome of 4.2Mbp (NCBI accession number CP034247). Gene annotation revealed 3869 genes in the genome, including 3744 protein-coding genes, 73 rRNAs/tRNAs/ncRNAs, and 52 pseudogenes. We compared the resistance determinants of SUE with six other public *Elizabethkingia anophelis* genomes with antibiotic-resistant profiles provided (Table 3, Supplementary Table S2). The Minimum Inhibitory Concentration (MIC) indicated three of them were resistant to TMP/SMX and to ciprofloxacin (Table 3, Supplementary Table S3), while the other four were sensitive to these agents.

**Figure 1:**
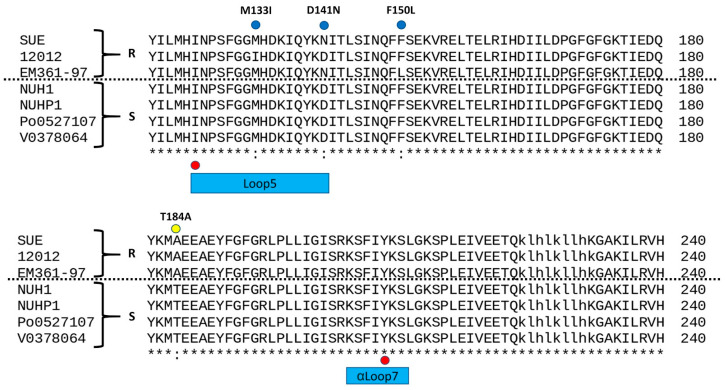
Sequence alignment of seven *E anopheles* DHPS orthologs. Residues marked in red dots are loci of known sulfa resistance mutations, and blue/yellow dots indicate those residues mutated in the current study. Loop1, loop2, loop5, and helix αLoop7 are boxed in bars with different colors.

**Figure 2:**
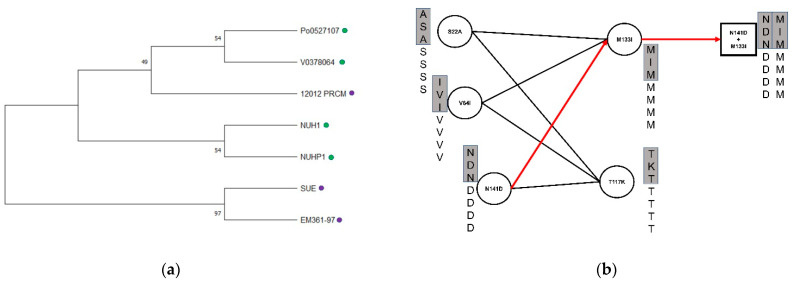
(**a**) Phylogeny of *folp* (DHPS) of seven *E. anopheles* strains. Green and purple circles indicate sensitive and resistance strains, respectively; (**b**) Identification of epi-static mutation via combinatorial enumeration. Each circle indicates a mutation point of seven *E. anopheles*, including three of them boxed in a gray bar, which were resistant to TMP/SMX. A line drawn between two circles represents a feasible mutation combination. An example of a combination (D141N+M133I) is shown on the right.

Genomic analysis revealed nine mutations in *folp* possibly related to TMP/SMX resistance (Figure 1), which were completely nonoverlapped with seventeen known *folp* mutations in the literature (Supplementary Table S4). Phylogeny reconstruction of seven *folp* amino acid sequences revealed their similarity and clusters (Figure 2a). Although two resistant strains, SUE and EM361-97, were clustered in the same clade, the resistant strain 12012 was clustered with other sensitive strains. This implies that the entire *folp* sequence is not sufficient for distinguishing resistant from susceptible strains, probably owing to many neutral mutations unrelated to the resistance.

**Figure 3:**
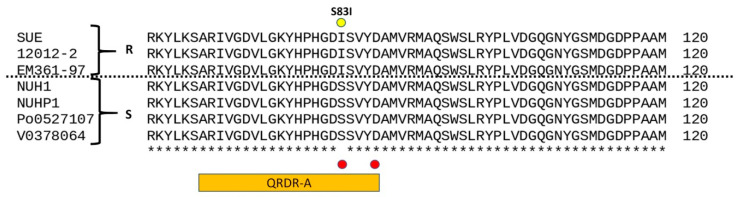
Sequence alignment of seven *E anopheles* DNA gyrase subunit A orthologs. Residues marked in red dots are sites of common quinolone drug resistance mutations. The yellow dot indicates a specific residue mutated in the current study, which was Isoleucine in the three quinolone-resistant strains and Serine in the four quinolone-sensitive strains QRDR-A are boxed in an orange bar.

We then assessed the statistical significance of each mutation associated with TMP/SMX resistance. One mutation, T184A, was significantly associated (*p* = 0.0286, Fisher’s exact test), while the others only showed weak or no correlation. In order to identify weakly associated variants yet with synergistic effects (i.e., epistatic interactions), we investigated their combinations associated with resistance (Figure 2b). All possible combinations of nine mutations were enumerated and validated for the ability to distinguish resistant strains from sensitive strains. Eleven combinations were found to be associated with TMP/SMX resistance. Among them, the M133I+D141N mutational combination was located on loop 5 (Figure 3), in which known mutations have been reported. Although the T184A mutation exhibited statistical significance, the locus was not within the conserved loop.

Similarly, a comparison of the DNA gyrase subunit A (GyrA) orthologous in the seven *E anopheles* genomes was revealed on mutation S83I located within the quinolone-resistance determining region (QRDR) (Figure 3). The amino acid changes were the same as those reported for fluoroquinolone resistance in *Proteus mirabilis* and *Proteus stuartii*, as confirmed experimentally by site-directed mutagenesis.

## 3. Discussion

*Elizabethkingia* species, previously named *Chryseobacterium*, are gram-negative, aerobic, nonmotile rod-shaped bacteria, and were re-identified by Kim, 2005 [1]. *Elizabethkingia anophelis* was first discovered in 2011 from the midgut of mosquitoes in Africa, and the first clinical infection, a case with neonatal meningitis, was reported in the same year in the Central Africa Republic [2,19].

The first nosocomial outbreak of *E. anophelis* infection occurred in a Singapore intensive care unit in 2012, and another outbreak occurred in hospitals in Wisconsin, USA, in 2015–2016 [5,6]. Most cases presented as blood stream infection and nosocomial pneumonia. Other infections including neonatal meningitis, biliary tract infection, septic arthritis, infective ascites, and eye infection were also reported [6,7,8]. The majority of patients with *E. anophelis* infection had previous underlying disease, including malignancy, diabetes mellitus, and recent major operation. [10] Most cases were nosocomial infection, and community-acquired disease accounted for a small proportion (<20% in HongKong), although the Wisconsin outbreak had a community infection rate of 84.6%. [6,10,18] Mortality and morbidity rates were relatively high among patients infected with *E. anophelis*, due to its broad antibiotic resistance. A cases series in Hong Kong, 2016, found a mortality rate of around 24–30%, and during the outbreak in Wisconsin, USA, the mortality rate in 30 days was 18.2% [6,18].

This is the first literature review for *Elizabethkingia* intra-abdominal infection. In our case series of *Elizabethkingia* intra-abdominal infection, infected ascites, biliary tract infection, postoperative peritonitis, and intra-abdominal abscess formation were found. However, in our review of the literature, no enterocolitis was found and no patients presented with diarrhea. For *Elizabethkingia* intra-abdominal infections, host factor was an important factor. Overall, 88.9% patients had underlying disease, indicating that *Elizabethkingia* species does not tend to infect healthy, immunocompetent individuals, which is in line with previous studies [9,10]. Diseases in infected patients included type II diabetes mellitus, hypertension, liver cirrhosis, end-stage kidney disease, and postliver transplantation, which are known to increase susceptibility to nosocomial infection [18,20].

In *Elizabethkingia* intra-abdominal infection, indwelling catheter, stenting, and recent operation were also important risk factors, and removal of the prosthesis plays an important role in successful treatment. In our literature review, two-thirds (66.7%) of our patients had indwelling catheter or stent, including one PTBD, two biliary stent, and three CAPD tube. Previous studies on blood stream infection also found catheter use was a significant factor for *Elizabethkingia* blood-stream infection [18]. Among the cases with CAPD peritonitis, infection was finally brought under control in two out of three cases after removal of the CAPD tube, suggesting that the removal of the catheter may be an important factor in successful treatment [15,16,17]. In addition to indwelling catheter or stent, recent operation or invasive procedure was also a significant risk factor for *Elizabethkingia* intra-abdominal infection. In our literature review, one patient had recent liver transplantation from a living donor within one month and the other patients had peritonitis just after medication termination of pregnancy [14,17]. The first outbreak in Singapore was noted in a surgical intensive care unit and in a cardiothoracic intensive care unit; three out of five patients were found to have *E. anophelis* infection after surgery [5]. Therefore, *Elizabethkingia* infection may be an important pathogen in postoperation infection and nosocomial infection.

*Elizabethkingia anophelis* caused the majority of *Elizabethkingia* infection, but true prevalence was underestimated because of misidentification. Most *Elizabethkingia anophelis* was misidentified as *E. meningoseptica*, by Vitek 2 and MOLDI-ToF [3,4,21]. *E. anophelis*, which was identified by 16s rRNA, accounted for 96.2% of *Elizabethkingia* infections in Singapore [3]. In another study in Korea, *E. anopheles*, re-identified by 16s rRNA sequencing, caused 59.3% of clinical *Elizabethkingia* infections [4]. In the study in Singapore during the period of 2009–2017, 76/79 of (96.2%) *E. anophelis* infections were misidentified as *E. meningoseptica* infections by MOLDI-ToF (bioMérieux) [3]. In a study by *Lin.* et al. in Taiwan, MALDI-TOF with knowledge base v 2.0/3.0 and Vitek 2 compact could only identify 26.5% *Elizabethkingia* species correctly when using 16s rRNA sequencing as the gold standard, and misidentified most *Elizabethkingia* species as *Elizabethkingia meningoseptica* [21]. In our case series, most cases with *E. meningoseptica* intra-abdominal infection were identified by the Vitek 2 compact, and misidentification was shown to be a problem. Misidentification can be improved by using 16s rRNA as the current standard and using the MOLDI-ToF system with change in databases and inclusion of mass spectra from seven *E. anophelis* isolates or SARAMIS database [4,18].

*Elizabethkingia anophelis* has exhibited broad antibiotic resistance; fluoroquinolones, TMP/SMX and piperacillin/tazobactam have been used as the first-line therapy [9,11]. However, there is large variability in susceptibility to fluoroquinolones (9.8%~70%), including ciprofloxacin and levofloxacin [11,22,23]. *Elizabethkingia* infection with resistance to fluoroquinolone can cause increased mortality [24]. Most resistance to fluoroquinolone in *E. anophelis* was caused by mutations in quinolone-resistance determining regions (QRDR), i.e., a single amino acid alteration in DNR gyrase or DNA topoisomerase IV [25,26]. Most common QRDR were noted in GyrA, including Ser83Ile, Ser83Arg [11,25,26]. Lin also reported other nonsynonymous alteration sites in the QRDR: two in GyrA (positions 95 and 102), and three in GyrB (positions 425, 452, and 470) [11]. Asp87Asn in GyrA was also reported in *E. miricola* [25]. In a study by Ming-Jr Jian, a 12.7-fold increase in the fluoroquinolone-related efflux pump AcrB was noted in fluoroquinolone-resistant *Elizabethkingia anophelis* strains, which may play a role in fluoroquinolone resistance [26]. Currently, no mutations in ParC or ParE have been reported in *Elizabethkingia* species. Further studies on other possible mechanisms for resistance are required to gain a more detailed understanding of quinolone resistance in *Elizabethkingia* species, such as efflux pump, drug-modifying enzyme, or plasmid mediated quinolone resistance [27].

There is limited information on resistance to Trimethoprim/sulfamethoxazole in *Elizabethkingia* species. Positive *dfr*A12, *sul* I and *sul* II gene in *Elizabethkingia* with resistance to TMP/SMX was found by PCR in a previous study [28]. The *Sul* I gene may be associated with integron, but no type I nor type II integron was noted in their study. However, there were still several strains with resistance to TMP/SMX that showed a negative finding in a previous study, suggesting other possible mechanisms than *sul* I, II, and *dfr* A1-12. We reported mutations in *folP*, including T184A, M133I and D141N, which may be associated with TMP/SMX resistance. However, as in previous studies, these mutations were not present in all resistant strains. There may be multiple other mutations and mechanisms involved in TMP/SMX resistance in *Elizabethkingia*, and thus, further investigation is needed.

## 4. Materials and Methods

### 4.1. Literature Review

We searched the English-language medical literature using PubMed/MEDLINE and Google Scholar from 1990 to 2019, using the following keywords: *Elizabethkingia meningoseptica, Elizabethkingia anophelis*, intra-abdominal infection, ascites infection, biliary infection. The references of articles found using this search were also reviewed to identify other potential cases that were not located using the search terms.

### 4.2. Whole Genome Sequencing and Bioinformatics Analysis

The SUE genome was deeply sequenced using Nanopore long-read sequencing and Illumina short-read sequencing (Supplementary Table S2). Adaptor sequences left in long reads were trimmed using Porechop. The remaining reads were hybrid assembled by Unicycler (v0.4.7) into a 4.2 Mbp circular genome. Protein-coding genes, coding and noncoding RNAs in the chromosomes, and plasmids were annotated by NCBI PGAP pipeline. Antibiotic-resistant genes were predicted by aligning protein-coding genes against the Comprehensive Antibiotic Resistance Database (CARD) using Diamond. Only ARGs with alignment coverage greater than 90% were retained. Efflux pumps were excluded from ARG analysis.

Multiple sequence alignment of DHPS (*folP*) and GyrA of the seven *E anopheles* genomes were carried out by MEGA X in order to identity mutation loci. The multiple sequence alignment of DHPS was also used to generate a phylogeny tree by MEGA X. The nine mutations within DHPS were tested for association with sulfa resistance via Fisher’s exact test. All possible combinations of these nine mutations (2^9^) were tested for correlation with TMP resistance by solving a combinatorial problem known as the *minimum test collection*; that is, only the combinations able to distinguish resistant strains from sensitive strains are retained as solutions.

## 5. Conclusions

*Elizabethkingia* intra-abdominal infection is a relatively uncommon condition to which patients with underlying disease are more susceptible. In our review of intra-abdominal infections, infective ascites, biliary tract infection, CAPD peritonitis, and postprocedural peritonitis were reported. Indwelling catheter and recent invasive procedure may be important risk factors, and removal of catheter was shown to be key to successful treatment. *Elizabethkingia anophelis* infection rates may be underestimated due to misidentification. We also conducted a genomic analysis to investigate antibiotic resistance genes in *Elizabethkingia anophelis*. *Fol p* was found to be associated with SMX/TMP resistance, and *Gyr* A was related to fluroquinolone resistance. However, these mutations were not present in all resistant strains, and multiple mutations may be associated with resistance. Further studies on the mechanism of resistance to SMX/TMP in *Elizabethkingia* infection are needed.

## Figures and Tables

**Table 1:** Clinical features of intra-abdominal infection with *Elizabethkingia spp.*

No.	Location	Age	Sex	Underlining Disease	Clinical Presentation	Culture- Positive Specimens	*Elizabethkingia* Speices	Method for Identification	References
1	Taichung, Taiwan	69	M	Autoimmune protein losing enteropathy, with ascites, pleural effusion	Fever, abdominal distention, Spontaneous bacterial peritonitis	Blood and ascites	*E. anophelis*	Whole genome sequencing	NA
2	Wisconsin, USA	84	M	Chronic HCV infection, cirrhosis, Type 2 DM, alcohol abuse	Abdominal distention, fever, Suspected spontaneous bacterial peritonitis	blood and ascites	*E. anophelis*	Verigene system /MALDI-ToF MS	[6]
3	Saudi Arabia	55	F	Type 2 DM, liver cirrhosis, s/p liver transplantation. with post-transplant anastomotic biliary stricture and bile leakage, s/p PTBD	RUQ abdominal pain, no fever. Intra-abdominal infection with subphrenic fluid accumulation	Bile *	*E. meningoseptica*	NA	[14]
4	Hongkong, China	52	M	biliary pancreatitis, recurrent pyogenic cholangitis, cirrhosis with biliary stent	Acute cholangitis	Blood	*E. meningoseptica*	16S rRNA gene sequence analysis	[18]
5	Hongkong, China	89	F	Hypertension, Atrial fibrillation, painless obstructive jaundice on palliative stenting	Biliary tract infection with sepsis	Blood	*E. meningoseptica*	16S rRNA gene sequence analysis	[18]
6	Tamil Nadu, India	72	M	Type 2 DM, hypertension, ESRD under CAPD for 22 months	Abdominal pain, and cloudy CAPD fluid. CAPD peritonitis	CAPD fluid	*E. meningoseptica*	Vitek-2 compact	[15]
7	Taipei, Taiwan	54	F	ESRD under CAPD for eight years	Turbid CAPD fluid, abdominal pain and fever. CAPD peritonitis with Tenckhoff tube infection	CAPD fluid	*E. meningoseptica (previously Chryseobacterium meningoseptica)*	API and Vitek test system	[16]
8	New Delhi, India	8	F	ESRD since 18 m/o, under CAPD for six years	CAPD peritonitis	CAPD fluid	*Elizabethkingia meningoseptica*	Vitek system and Vitek AST-N090 card	[17]
9	New Delhi, India	23	F	s/p Medical Termination of Pregnancy by suction and evacuation for suspected blighted ovum or missed abortion	Postposture fever and bleeding. Peritonitis, secondary to uterine perforation	blood culture * 2 set	*Elizabethkingia meningoseptica*	Vitek system and Vitek AST-N090 card	[17]

Under immunosuppressant with oral tacrolimus 1mg, oral mycophenolate mofetil 500 mg, and oral prednisolone 5 mg; * from PTBD; NA, nonavailable; HCV, Hepatitis C virus; type 2 DM, type 2 diabetes mellitus; PTBD, percutaneous biliary drainage; ESRD, End-stage renal disease; CAPD, Continuous Ambulatory Peritoneal Dialysis.

**Table 2:** Treatment for Intra-abdominal infection with *Elizabethkingia spp.*

No.	*Elizabethkingia spp.*	Clinical Presentation	Antibiotic Susceptibility (Susceptible Drugs)	Antibiotic Use	Removal of Catheter	Survival
1	*E. anophelis*	Spontaneous bacterial peritonitis	Pipercacillin/tazobactam, Cefepime, Cefoperazone/sulbactam; TMP/SMX *	TMP/SMX+ Levofloxacin, then Piperacillin/tazobactam	No catheter	Expired
2	*E. anophelis*	Suspected spontaneous bacterial peritonitis	Ciprofloxacin, Piperacillin/tazobactam, TMP/SMX, Cefepime	Ciprofloxacin and Piperacillin/tazobactam	No catheter	Survived
3	*E. meningoseptica*	Intra-abdominal infection with subphrenic fluid accumulation	Ciprofloxacin, Minocycline, Tigecycline, TMP/SMX	Ciprofloxacin and metronidazole	NA	Survived
4	*E. meningoseptica*	Acute cholangitis	NA	Levofloxacin and metronidazole	NA	Survived
5	*E. meningoseptica*	Biliary tract infection with sepsis	NA	Levofloxacin	NA	Survived
6	*E. meningoseptica*	CAPD peritonitis	Cefoperazone/sulbactam, Ciprofloxacin, Levofloxacin, Minocycline, TMP/SMX	PO TMP/SMX + IV Cefoperazone/sulbactam, then shift to PO Minocycline + IV Cefoperazone/sulbactam	Removal of CAPD tube.	Survived
7	*E. meningoseptica (previously Chryseobacterium meningoseptica)*	CAPD peritonitis with Tenckhoff tube infection	Gentamicin, Ciprofloxacin, Piperacillin-tazobactam, Levofloxacin	Piperacillin-tazobactam, then shift to Levofloxacin	Removal of CAPD tube.	Survived
8	*E. meningoseptica*	CAPD peritonitis	Cefoperazone-sulbactam and nalidixic acid	Cefoperazone-sulbactam	NA	Survived
9	*E. meningoseptica*	Peritonitis, secondary to uterine perforation	TMP/SMX	TMP/SMX, Piperacillin- tazobactam, amikacin, teicoplanin and metronidazole	No catheter	Survived

* TMP/SMX-susceptible at first but became resistant in repeated blood culture and ascites culture; NA, nonavailable; CAPD, Continuous Ambulatory Peritoneal Dialysis; TMP/SMX, Trimethoprim/sulfamethoxazole.

**Table 3:** Comparison of seven *Elizabethkingia anophelis* genomes and antibiotic resistance.

Strains	Genome Size	Genes	Sequencing Technology	TMP/SMX	Quinolone	Status	Assembly Number
SUE	4,201,198 bp	3869	Nanopore; Illumina MiSeq	160 R	≥4 R	Circ.	GCA_014702245.1
12012	4,023,312 bp	3700	Illumina MiSeq	>2/38 R	>2 R	Linear	GCA_001482795.1
EM361-97	4,077,699 bp	3752	Illumina HiSeq	>4/76 R	>2 R	Linear	GCA_001703835.1
NUH1	4,334,661 bp	4031	Illumina MiSeq	S	S	Linear	GCA_000495995.1
NUHP1	4,369,828 bp	4034	Illumina	S	S	Linear	GCA_000495935.2
Po0527107	4,032,057 bp	3717	Illumina HiSeq-2000	S	S	Linear	GCA_000689515.1
V0378064	4,036,754 bp	3804	Illumina HiSeq-2001	S	I	Linear	GCA_000689455.1

S: susceptible; I: intermediate; R: resistance.

## Data Availability

All the sequencing data have been deposited in GenBank under BioProject ID no. PRJNA507867.

## Supplementary Materials

The following are available online at https://www.mdpi.com/2079-6382/10/2/173/s1, Table S1: Summary of sequencing and assembly statistics, Table S2: Genome features of strains analysed in the study, Table S3: Antibiotic susceptibility profile of strains analyzed in the study, Table S4: Identified folp mutations in SUE, Table S5: folP mutation combinations in SUE.
